# Editorial: Different cell death modes in cancer treatment

**DOI:** 10.3389/fphar.2023.1291585

**Published:** 2023-09-27

**Authors:** Zhaoshi Bai, Jie Dou, Tareq Saleh, Jingwen Xu, Wufu Zhu

**Affiliations:** ^1^ Jiangsu Cancer Hospital and Jiangsu Institute of Cancer Research and the Affiliated Cancer Hospital of Nanjing Medical University, Nanjing, Jiangsu, China; ^2^ School of Life Science and Technology, China Pharmaceutical University, Nanjing, Jiangsu, China; ^3^ Department of Pharmacology and Public Health, Faculty of Medicine, The Hashemite University, Zarqa, Jordan; ^4^ Guangdong Pharmaceutical University, Guangzhou, China; ^5^ Jiangxi Science and Technology Normal University, Nanchang, China

**Keywords:** cell death modes, mechanism, interaction, anticancer drugs, drug discovery

Anticancer drugs exert effects by inducing various types of cell death, including apoptosis, necroptosis, ferroptosis, pyroptosis, parthanatos, entotic cell death, autophagy, cellular senescence, and mitotic catastrophe (Galluzzi et al., 2018; Wang et al., 2022). These cell death modes are usually not presented separately, resulting in the complex interactions (including synergistic, complementary, additive, even competitive or antagonistic effects) among them during cancer treatment (Qi et al., 2022; Zhao et al., 2022). Therefore, in-depth understanding of the molecular mechanisms underlying drug-induced different cell death modes and the interactions among these cell death modes, has a positive significance for the research and development of new anticancer drugs and treatment strategies. In the current Research Topic “*Different Cell Death Modes in Cancer Treatment*,” we attempted to collect the most-recent progress made in cell death modes in cancer treatment. In total, after being peer-reviewed, seven manuscripts, composed with four research articles and three review articles authored by 49 researchers worldwide, were successfully accepted for publication.

Currently, most drugs are able to exert potent anticancer effects by inducing apoptosis (Nossing and Ryan, 2022). Chen et al. reported that Yiwei decoction (YWD), a traditional Chinese medicine (TCM) formula, can promote apoptosis of gastric cancer cells through spleen-derived exosomes, thereby supporting the use of YWD-treated exosomes as a new approach for the clinical treatment of gastric cancer. However, due to the fact that apoptosis is usually codetermined by dozens of proapoptotic and antiapoptotic genes/proteins, multidrug resistance (MDR) mediated by apoptosis resistance is more common than that mediated by other factors (Bueschbell et al., 2022). Thus, it is very attractive to avoid the apoptosis resistance pathway by inducing non-apoptotic cell death modes. Yang et al. reviewed the current status of research on the molecular mechanisms and targets through quercetin-mediated non-apoptotic mode of cancer cell death. Autophagy is a lysosome-mediated catabolic process of cell adaptation to metabolic and environmental stress and plays a complex effect in cancer treatment (Manzoor et al., 2022). Zhang et al. reviewed the roles and underlying mechanisms of Tripartite motif (TRIM) family proteins in tumorigenesis and progression. Further, they pointed out that TRIMs may be the core components of autophagy or part of the autophagy-induced pathway in cancer treatment. Paraptosis is an alternative cell death pathway characterized by vacuolation and damage to the endoplasmic reticulum and mitochondria (Hanson et al.). Hanson et al. presented a systematic review of the growth, challenges, and future perspectives of paraptosis research in cancer treatment, which will help to develop potential therapy and combat chemoresistance in various cancer. Cuproptosis, a newly defined regulation form of cell death, is mediated by the accumulation of copper ions in cells and related to protein lipoacylation (Hadian and Stockwell, 2023; Wu et al., 2023). Ma et al. found that an eight-lncRNA signature (TSPOAP1-AS1, AC107464.3, AC006449.7, LINC00324, COLCA1, HAGLR, MIR4435-2HG, and NKILA) linked to cuproptosis was identified, which may improve lung adenocarcinoma management strategies. Xu et al. reported that cuproptosis-related prognostic 2-lncRNAs signature (BCCuS) may be useful in predicting the prognosis, biological characteristics, and appropriate treatment of breast cancer patients. In addition, the induction of immunogenic cancer cell death has become one of the aims of drugs because it would allow the immune system to contribute through a “bystander effect” to the eradication of therapy-resistant cancer cells and cancer stem cells (Kroemer et al., 2022). Based on a bibliometric analysis, Zhou et al. provided an intuitive approach to investigate the research trends and hotspots concerning immunogenic cell death in cancer treatment.

In conclusion, an increasing number of cell death modes have attracted attention in recent years. Each cell death mode has unique advantages and disadvantages, and fully utilizing their advantages and avoiding their disadvantages has positive significance in cancer treatment.
